# 7^th^ Brazilian Guideline of Arterial Hypertension: Chapter 10 - Hypertension in Children and Adolescents

**DOI:** 10.5935/abc.20160160

**Published:** 2016-09

**Authors:** MVB Malachias, V Koch, FC Colombo, ACS Silva, ICB Guimarães, PK Nogueira

## Epidemiological context and importance of hypertension in pediatrics

Arterial hypertension was identified as the major source of combined mortality and morbidity, representing 7% of global disability-adjusted life years.^1^ The adoption of the BP definitions and normalization of the "National High Blood Pressure Education Program" (NHBPEP) 2004^2^ has standardized the BP classification in the pediatric population. The percentage of children and adolescents diagnosed with AH is estimated to have doubled in the past two decades. The current prevalence of AH in the pediatric population is around 3% to 5%,^3-5^ while that of PH reaches 10% to 15%,^3,4,6,7^ and such values are mainly attributed to the large increase in childhood obesity.^8^ The etiology of pediatric AH can be either secondary, most often associated with nephropathies, or primary, attributed to genetic causes with environmental influence, predominating in adolescents.

Pediatric AH is usually asymptomatic, but as many as 40% of hypertensive children have LVH at the initial diagnosis of AH. Although oligosymptomatic in childhood, LVH is a precursor of arrhythmias and HF in adults.^9^ In addition, pediatric AH is associated with the development of other changes in target organs, such as increased carotid IMT, arterial compliance reduction, and retinal arteriolar narrowing. Early diagnosis and treatment of childhood AH are associated with a lower risk for AH and for increased carotid atheromatosis in adult life.^10^ Therefore, periodical BP measurements in children and adolescents are recommended, even contradicting the U.S. Preventive Services Task Force's suggestion, which considers the evidence of benefits of primary AH screening in asymptomatic children and adolescents insufficient to prevent CVD in childhood or adulthood.^11^

## Definitions and diagnosis

### Definition and etiology

Children and adolescents are considered hypertensive when SBP and/or DBP are greater than or equal to the 95^th^ percentile for age, sex and height percentile, on at least three different occasions.^2^ Prehypertension in children is defined as SBP/DBP ≥ the 90^th^ percentile < the 95^th^ percentile, and in adolescents as BP levels ≥ 120/80 mm Hg and < the 95^th^ percentile. Stage 1 AH is considered for readings between the 95^th^ percentile and the 99^th^ percentile plus 5 mm Hg, while stage 2 AH, for readings > stage 1. The height percentiles can be obtained by using Centers for Disease Control and Prevention's (CDC) growth charts.^12^ In addition, normal and high BP levels for children and adolescents are available in mobile apps, such as PA Kids and Ped(z).

In the pediatric population, WCH and MH can be diagnosed based on established normality criteria for ABPM.^13^

After a detailed clinical history and physical examination, children and adolescents considered hypertensive should undergo investigation. The younger the child, the greater the chance of secondary AH. Parenchymal, renovascular and obstructive nephropathies account for approximately 60-90% of the cases, and can affect all age groups (infants, children and adolescents), being more prevalent in younger children with higher BP elevations. Endocrine disorders, such as excessive mineralocorticoid, corticoid or catecholamine secretion, thyroid diseases and hypercalcemia associated with hyperparathyroidism, account for approximately 5% of secondary AH cases. Coarctation of the aorta is diagnosed in 2% of the cases, and 5% of secondary AH cases are attributed to other etiologies, such as adverse effects of vasoactive and immunosuppressive drugs, steroid abuse, central nervous system changes, and increased intracranial pressure.

Primary AH is more prevalent in overweight or obese children and adolescents with family history of AH. Currently, primary AH seems to be the most common form of AH in adolescence, being, however, a diagnosis of exclusion, and, in that population, secondary causes should be investigated whenever possible.

### Diagnosis

#### Method for BP measurement

Measuring BP in children is recommended at every clinical assessment after the age of 3 years, abiding by the standards for BP measurement.^2^ Children under the age of 3 years should have their BP assessed on specific situations.^2,14^ For BP measurement, children should be calm and sitting for at least 5 minutes, with back supported and feet on the floor, having refrained from consuming stimulant foods and beverages. The BP should be taken at heart level on the right arm, because of the possibility of coarctation of the aorta. Table 1 shows the specific recommendations for auscultatory BP measurement in children and adolescents. Whenever BP is high on the upper limbs, SBP should be assessed on the lower limbs. Such assessment can be performed with the patient lying down, with the cuff placed on the calf, covering at least two-thirds of the knee-ankle distance. The SBP reading on the leg can be higher than that on the arm because of the distal pulse amplification phenomenon. A lower SBP reading on the leg as compared to that on the arm suggests coarctation of the aorta.

**Table 1:** Specific recommendations for BP measurement in children and adolescents

• Auscultatory method.
• Use 1^st^ Korotkoff sound for SBP, and 5^th^ Korotkoff sound for DBP.
• When using the oscillometric device, it requires validation.
• Detection of AH by use of the oscillometric device requires confirmation with auscultation.
• Use appropriate cuff size; air bag width: 40% of arm circumference in the middle point between the acromion and olecranon, and air bag length: 80-100% of arm circumference.
• Conditions under which children < 3 years old should have BP measured: neonatal intensive care; congenital heart diseases, kidney diseases, treatment with drugs known to raise BP, and evidence of increased intracranial pressure.

**Figure 1:**
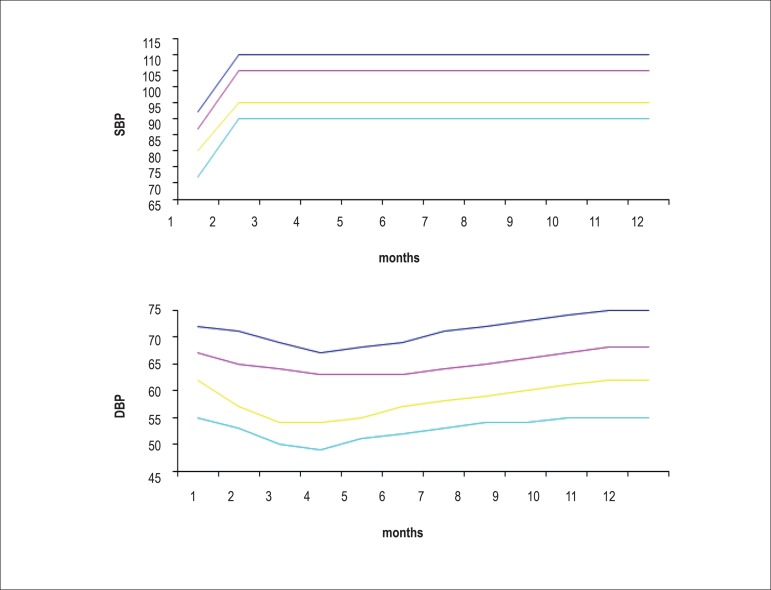
Blood pressure levels for boys, from birth to the age of 1 year^97^

**Figure 2:**
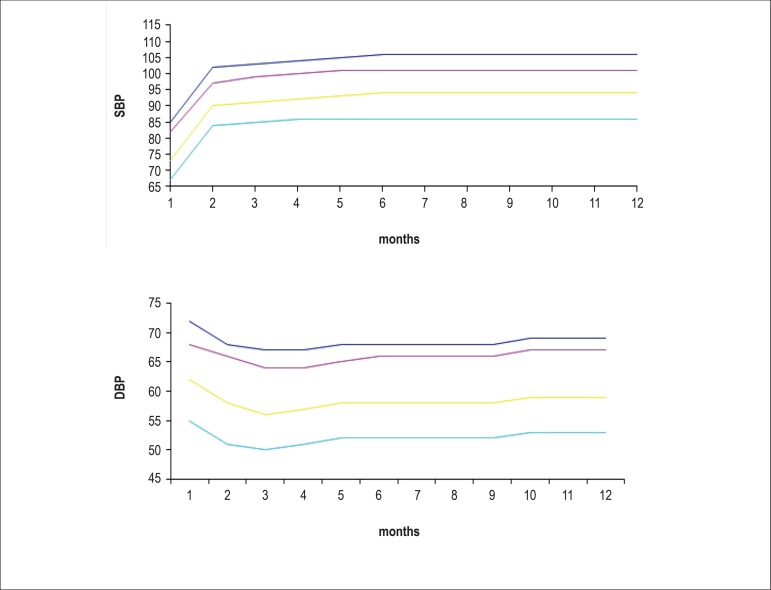
Blood pressure levels for girls, from birth to the age of 1 year^97^

**Note:** Adolescents with BP ≥ 120/80 mm Hg should be considered prehypertensive, even if the 90^th^ percentile value is greater than that. This can occur for SBP in patients older than 12 years, and for DBP in patients older than 16 years.

Tables 2 and 3 show the BP percentiles by sex, age and height percentile. Figures 1 and 2 show BP values for boys and girls, respectively, from birth to the age of 1 year based on data from the Report of the Second Task Force on Blood Pressure Control in Children - 1987.^15^

**Table 2:** Blood pressure levels for boys by age and height percentile^2^

	BP	SBP (mm Hg)							DBP (mm Hg)						
Age	percentile	← Percentile of Height →							← Percentile of Height →						
(Year)		5^th^	10^th^	25^th^	50^th^	75^th^	90^th^	95^th^	5^th^	10^th^	25^th^	50^th^	75^th^	90^th^	95^th^
1	50^th^	80	81	83	85	87	88	89	34	35	36	37	38	39	39
	90^th^	94	95	97	99	100	102	103	49	50	51	52	53	53	54
	95^th^	98	99	101	-103	104	106	106	54	54	55	56	57	58	58
	99^th^	105	106	108	110	112	113	114	61	62	63	64	65	66	66
2	50^th^	84	85	87	88	90	92	92	39	40	41	42	43	44	44
	90^th^	97	99	100	102	104	105	106	54	55	56	57	58	58	59
	95^th^	101	102	104	106	108	109	110	59	59	60	61	62	63	63
	99^th^	109	110	111	113	115	117	117	66	67	68	69	70	71	71
3	50^th^	86	87	89	91	93	94	95	44	44	45	46	47	48	48
	90^th^	100	101	103	105	107	108	109	59	59	60	61	62	63	63
	95^th^	104	105	107	109	110	112	113	63	63	64	65	66	67	67
	99^th^	111	112	114	116	118	119	120	71	71	72	73	74	75	75
4	50^th^	88	89	91	93	95	96	97	47	48	49	50	51	51	52
	90^th^	102	103	105	107	109	110	111	62	63	64	65	66	66	67
	95^th^	106	107	109	111	112	114	115	66	67	68	69	70	71	71
	99^th^	113	114	116	118	120	121	122	74	75	76	77	78	78	79
5	50^th^	90	91	93	95	96	98	98	50	51	52	53	54	55	55
	90^th^	104	105	106	108	110	111	112	65	66	67	68	69	69	70
	95^th^	108	109	110	112	114	115	116	69	70	71	72	73	74	74
	99^th^	115	116	118	120	121	123	123	77	78	79	80	81	81	82
6	50th	91	92	94	96	98	99	100	53	53	54	55	56	57	57
	90^th^	105	106	108	110	111	113	113	68	68	69	70	71	72	72
	95^th^	109	110	112	114	115	117	117	72	72	73	74	75	76	76
	99^th^	116	117	119	121	123	124	125	80	80	81	82	83	84	84
7	50^th^	92	94	95	97	99	100	101	55	55	56	57	58	59	59
	90^th^	106	107	109	111	113	114	115	70	70	71	72	73	74	74
	95^th^	110	111	113	115	117	118	119	74	74	75	76	77	78	78
	99^th^	117	118	120	122	124	125	126	82	82	83	84	85	86	86
8	50^th^	94	95	97	99	100	102	102	56	57	58	59	60	60	61
	90^th^	107	109	110	112	114	115	116	71	72	72	73	74	75	76
	95^th^	111	112	114	116	118	119	120	75	76	77	78	79	79	80
	99^th^	119	120	122	123	125	127	127	83	84	85	86	87	87	88
9	50^th^	95	96	98	100	102	103	104	57	58	59	60	61	61	62
	90^th^	109	110	112	114	115	117	118	72	73	74	75	76	76	77
	95^th^	113	114	116	118	119	121	121	76	77	78	79	80	81	81
	99^th^	120	121	123	125	127	128	129	84	85	86	87	88	88	89
10	50^th^	97	98	100	102	103	105	106	58	59	60	61	61	62	63
	90^th^	111	112	114	115	117	119	119	73	73	74	75	76	77	78
	95^th^	115	116	117	119	121	122	123	77	78	79	80	81	81	82
	99^th^	122	123	125	127	128	130	130	85	86	86	88	88	89	90
11	50^th^	99	100	102	104	105	107	107	59	59	60	61	62	63	63
	90^th^	113	114	115	J17	119	120	121	74	74	75	76	77	78	78
	95^th^	117	118	119	121	123	124	125	78	78	79	80	81	82	82
	99^th^	124	125	127	129	130	132	132	86	86	87	88	89	90	90
12	50^th^	101	102	104	106	108	109	110	59	60	61	62	63	63	64
	90^th^	115	116	118	120	121	123	123	74	75	75	76	77	78	79
	95^th^	119	120	122	123	125	127	127	78	79	80	81	82	82	83
	99^th^	126	127	129	131	133	134	135	86	87	88	89	90	90	91
13	50^th^	104	105	106	108	110	111	112	60	60	61	62	63	64	64
	90^th^	117	118	120	122	124	125	126	75	75	76	77	78	79	79
	95^th^	121	122	124	126	128	129	130	79	79	80	81	82	83	83
	99^th^	128	130	131	133	135	136	137	87	87	88	89	90	91	91
14	50^th^	106	107	109	111	113	114	115	60	61	62	63	64	65	65
	90^th^	120	121	123	125	126	128	128	75	76	77	78	79	79	80
	95^th^	124	125	127	128	130	132	132	80	80	81	82	83	84	84
	99^th^	131	132	134	136	138	139	140	87	88	89	90	91	92	92
15	50^th^	109	110	112	113	115	117	117	61	62	63	64	65	66	66
	90^th^	122	124	125	127	129	130	131	76	77	78	79	80	80	81
	95^th^	126	127	129	131	133	134	135	81	81	82	83	84	85	85
	99^th^	134	135	136	138	140	142	142	88	89	90	91	92	93	93
16	50^th^	111	112	114	116	118	119	120	63	63	64	65	66	67	67
	90^th^	125	126	128	130	131	133	134	78	78	79	80	81	82	82
	95^th^	129	130	132	134	135	137	137	82	83	83	84	85	86	87
	99^th^	136	137	139	141	143	144	145	90	90	91	92	93	94	94
17	50^th^	114	115	116	118	120	121	122	65	66	66	67	68	69	70
	90^th^	127	128	130	132	134	135	136	80	80	81	82	83	84	84
	95^th^	131	132	134	136	138	139	140	84	85	86	87	87	88	89
	99^th^	139	140	141	143	145	146	147	92	93	93	94	95	96	97

**Table 3:** Blood pressure levels for girls by age and height percentile2

	BP	SBP (mm Hg)							DBP (mm Hg)						
Age	Percentile	← Percentile of Height →							← Percentile of Height →						
(Year)		5^th^	10^th^	25^th^	50^th^	75^th^	90^th^	95^th^	5^th^	10^th^	25^th^	50^th^	75^th^	90^th^	95^th^
1	50^th^	83	84	85	86	88	89	90	38	39	39	40	41	41	42
	90^th^	97	97	98	100	101	102	103	52	53	53	54	55	55	56
	95^th^	100	101	102	104	105	106	107	56	57	57	58	59	59	60
	99^th^	108	108	109	111	112	113	114	64	64	65	65	66	67	67
2	50^th^	85	85	87	88	89	91	91	43	44	44	45	46	46	47
	90^th^	98	99	100	101	103	104	105	57	58	58	59	60	61	61
	95^th^	102	103	104	105	107	108	109	61	62	62	63	64	65	65
	99^th^	109	110	111	112	114	115	116	69	69	70	70	71	72	72
3	50^th^	86	87	88	89	91	92	93	47	48	48	49	50	50	51
	90^th^	100	100	102	103	104	106	106	61	62	62	63	64	64	65
	95^th^	104	104	105	107	108	109	110	65	66	66	67	68	68	69
	99^th^	111	111	113	114	115	116	117	73	73	74	74	75	76	76
4	50^th^	88	88	90	91	92	94	94	50	50	51	52	52	53	54
	90^th^	101	102	103	104	106	107	108	64	64	65	66	67	67	68
	95^th^	105	106	107	108	110	111	112	68	68	69	70	71	71	72
	99^th^	112	113	114	115	117	118	119	76	76	76	77	78	79	79
5	50^th^	89	90	91	93	94	95	96	52	53	53	54	55	55	56
	90^th^	103	103	105	106	107	109	109	66	67	67	68	69	69	70
	95^th^	107	107	108	110	111	112	113	70	71	71	72	73	73	74
	99^th^	114	114	116	117	118	120	120	78	78	79	79	80	81	81
6	50^th^	91	92	93	94	96	97	98	54	54	55	56	56	57	58
	90^th^	104	105	106	108	109	110	111	68	68	69	70	70	71	72
	95^th^	108	109	110	111	113	114	115	72	72	73	74	74	75	76
	99^th^	115	116	117	119	120	121	122	80	80	80	81	82	83	83
7	50^th^	93	93	95	96	97	99	99	55	56	56	57	58	58	59
	90^th^	106	107	108	109	111	112	113	69	70	70	71	72	72	73
	95^th^	110	111	112	113	115	116	116	73	74	74	75	76	76	77
	99^th^	117	118	119	120	122	123	124	81	81	82	82	83	84	84
8	50^th^	95	95	96	98	99	100	101	57	57	57	58	59	60	60
	90^th^	108	109	110	111	113	114	114	71	71	71	72	73	74	74
	95^th^	112	112	114	115	116	118	118	75	75	75	76	77	78	78
	99^th^	119	120	121	122	123	125	125	82	82	83	83	84	85	86
9	50^th^	96	97	98	100	101	102	103	58	58	58	59	60	61	61
	90^th^	110	110	112	113	114	116	116	72	72	72	73	74	75	75
	95^th^	114	114	115	117	118	119	120	76	76	76	77	78	79	79
	99^th^	121	121	123	124	125	127	127	83	83	84	84	85	86	87
10	50^th^	98	99	100	102	103	104	105	59	59	59	60	61	62	62
	90^th^	112	112	114	115	116	118	118	73	73	73	74	75	76	76
	95^th^	116	116	117	119	120	121	122	77	77	77	78	79	80	80
	99^th^	123	123	125	126	127	129	129	84	84	85	86	86	87	88
11	50^th^	100	101	102	103	105	106	107	60	60	60	61	62	63	63
	90^th^	114	114	116	117	118	119	120	74	74	74	75	76	77	77
	95^th^	118	118	119 -121		122	123	124	78	78	78	79	80	81	81
	99^th^	125	125	126	128	129	130	131	85	85	86	87	87	88	89
12	50^th^	102	103	104	105	107	108	109	61	61	61	62	63	64	64
	90^th^	116	116	117	119	120	121	122	75	75	75	76	77	78	78
	95^th^	119	120	121	123	124	125	126	79	79	79	80	81	82	82
	99^th^	127	127	128	130	131	132	133	86	86	87	88	88	89	90
13	50^th^	104	105	106	107	109	110	110	62	62	62	63	64	65	65
	90^th^	117	118	119	121	122	123	124	76	76	76	77	78	79	79
	95^th^	121	122	123	124	126	127	128	80	80	80	81	82	83	83
	99^th^	128	129	130	132	133	134	135	87	87	88	89	89	90	91
14	50^th^	106	106	107	109	110	111	112	63	63	63	64	65	66	66
	90^th^	119	120	121	122	124	125	125	77	77	77	78	79	80	80
	95^th^	123	123	125	126	127	129	129	81	81	81	82	83	84	84
	99^th^	130	131	132	133	135	136	136	88	88	89	90	90	91	92
15	50^th^	107	108	109	110	111	113	113	64	64	64	65	66	67	67
	90^th^	120	121	122	123	125	126	127	78	78	78	79	80	81	81
	95^th^	124	125	126	127	129	130	131	82	82	82	83	84	85	85
	99^th^	131	132	133	134	136	137	138	89	89	90	91	91	92	93
16	50^th^	108	108	110	111	112	114	114	64	64	65	66	66	67	68
	90^th^	121	122	123	124	126	127	128	78	78	79	80	81	81	82
	95^th^	125	126	127	128	130	131	132	82	82	83	84	85	85	86
	99^th^	132	133	134	135	137	138	139	90	90	90	91	92	93	93
17	50^th^	108	109	110	111	113	114	115	64	65	65	66	67	67	68
	90^th^	122	122	123	125	126	127	128	78	79	79	80	81	81	82
	95^th^	125	126	127	129	130	131	132	82	83	83	84	85	85	86
	99^th^	133	133	134	136	137	138	139	90	90	91	91	92	93	93

For children/adolescents, ABPM is indicated to investigate WCH and MH, and to follow prehypertensive or hypertensive patients up.^13^ The prevalence of WCH has been reported as between 22% and 32%. The use of ABPM should be restricted to patients with borderline or mild AH, because patients with high office BP readings are more likely to be hypertensive.^16^

#### Anamnesis

A careful recollection of data on birth, growth and development, personal antecedents, and renal, urological, endocrine, cardiac and neurological diseases should be performed. The following patterns should be characterized: physical activity; dietary intake; smoking habit and alcohol consumption; use of steroids, amphetamines, sympathomimetic drugs, tricyclic antidepressants, contraceptives and illicit substances; and sleep history, because sleep disorders are associated with AH, overweight and obesity. In addition, family antecedents for AH, kidney diseases and other CVRF should be carefully assessed.

#### Physical examination

On physical examination, BMI should be calculated.^17^ Growth delay might suggest chronic disease, and persistent tachycardia might suggest hyperthyroidism or pheochromocytoma. Pulse decrease on the lower limbs leads to the suspicion of coarctation of the aorta. Adenoid hypertrophy is associated with sleep disorders. *Acantosis nigricans* suggests insulin resistance and DM. Abdominal fremitus and murmurs can indicate renovascular disease.^18^

#### Complementary tests

Laboratory and imaging tests are aimed at defining the etiology of AH (primary or secondary) and detecting TOD and CVRF associated with AH (Tables 4 and 5).^2,14^

**Table 4:** Initial investigation of children and adolescents with AH

Complete blood count
Renal function and electrolytes (including calcium, phosphorus and magnesium)
Fasting lipid panel
Plasma uric acid levels
Fasting glucose
Urinalysis and urine culture
Retinal exam
Chest X ray
ECG / Doppler echocardiography
Renal US with Doppler of renal arteries

**Table 5:** Complementary tests to confirm the etiology ofsecondary AH in children and adolescents

Measurement of urine electrolytes, proteinuria and urine creatinine
Plasma levels of renin (or plasma renin activity) and aldosterone, salivary cortisol test, PTH, TSH, free T4 and T3
Hemoglobin electrophoresis
Specific auto-antibodies: FAN, anti DNA, ANCA p, ANCA c
Urine catecholamines and metanephrines (or plasma metanephrine) and MIBG scintigraphy

MIBG: metaiodobenzylguanidine

Target-organ assessment should be performed in all children and adolescents with stage 1 and 2 AH. Sleep study by use of polysomnography or home respiratory polygraphy is indicated for children and adolescents with sleep disorders detected on anamnesis.^2^ To investigate secondary AH, see Chapter 12.

Table 5 shows some tests for children and adolescents suspected of having secondary AH.

## Therapeutic aspects

In children and adolescents with confirmed AH, therapeutic management is guided by the AH etiology definition, CV risk assessment, and TOD characterization.

### Nonpharmacological management

Nonpharmacological management should be introduced to all pediatric patients with BP levels above the 90^th^ percentile.^2^ (GR: IIa; LE: C). It includes body weight loss, a physical exercise program, and dietary intervention.^2^ Body weight reduction yields good results in the treatment of obese hypertensive children,^19^ similarly to physical exercise, which has better effect on SBP levels.^19^ Regular aerobic activity is recommended as follows: moderate-intensity physical exercise, 30-60 minutes/day, if possible, every day. Children with AH can practice resistance or localized training, except for weight lifting. Competitive sports are not recommended for patients with uncontrolled stage 2 AH.^20^ Dietary intervention can comprise sodium restriction,^21^ and potassium and calcium supplementation; the efficacy in that population, however, is yet to be proven.^22^

### Pharmacological management

Pharmacological therapy should be initiated for children with symptomatic AH, secondary AH, presence of TOD, types 1 and 2 DM, CKD and persistent AH nonresponsive to nonpharmacological therapy.^2^ (GR: IIa; LE: B). The treatment is aimed at BP reduction below the 95^th^ percentile in non-complicated AH, and BP reduction below the 90^th^ percentile in both complicated AH, characterized by TOD and comorbidities (DM, CKD), and secondary AH.^2^ (GR: IIa; LE: C). The treatment should begin with a first-line antihypertensive agent, whose dose should be optimized, and, if target BP level is not attained, other pharmacological groups should be added in sequence. A recent systematic review^23^ has identified neither a randomized study assessing the efficacy of antihypertensive drugs on TOD, nor any consistent dose-response relationship with any drug class assessed.

The adverse events associated with the use of antihypertensive agents for children and adolescents have been usually of mild intensity, such as headache, dizziness, and upper respiratory tract infections. All classes of antihypertensive drugs seem safe, at least in the short run.^23^ The only randomized, double-blind, controlled study, by Schaefer et al., comparing the efficacy and safety of drugs of parallel groups and assessing hypertensive children on enalapril or valsartan, has shown comparable results regarding the efficacy and safety of both drugs.^24^

In secondary AH, the antihypertensive drug choice should be in consonance with the pathophysiological principle involved, considering the comorbidities present. For example, non-cardioselective BBs should be avoided in individuals with upper airway reactivity, because of the risk for bronchospasm.^25^ In pregnancy, ACEIs and ARBs are contraindicated, because of their potential for fetal malformation.^26^ The use of those drugs for childbearing-age girls should be always accompanied by contraceptive guidance.^26,27^

For renovascular AH, of ACEIs or ARBs are indicated in association with vasodilators and DIUs. In cases of coarctation of the aorta, in the preoperative period, the initial drug is usually a BB. If the AH persists postoperatively, the BB can be maintained, replaced or associated with an ACEI or ARB. For AH associated with DM and CKD, an ACEI or ARB is initially used. The use of ACEI and ARB relaxes the efferent arteriole, reducing the glomerular capillary hydrostatic pressure, and posing a risk for AKI in situations of hypovolemia. Similarly, those drugs are contraindicated for patients with bilateral renal artery stenosis.^26-29^ For obese adults, ACEIs, ARBs, CCBs, BBs and DIUs are effective in reducing BP.^30^ In adults, ACEIs and ARBs seem to reduce the risk of developing DM and to increase insulin sensitivity.^31-33^

Table 6 shows the updated pediatric doses of the most frequently prescribed hypotensive agents to treat CAH.^2,27,28^

**Table 6:** Most frequently used oral drugs for management of pediatric chronic arterial hypertension2

Drug	Initial dose (mg/kg/dose)	Maximum dose (mg/kg/day)	Interval
Amlodipine (6-17 years)	0.1	0.5	24h
Nifedipine XL	0.25-0.5	3 (max:120 mg/day)	12-24h
Captopril			
Children	0.3-0.5	6	8h
Neonate	0.03-0.15	2	8-24h
Enalapril	0.08	0.6	12-24h
Losartan	0.7 (max: 50 mg/day)	1.4 (max: 100 mg/day)	24h
Propranolol	1-2	4 (max: 640 mg/day)	8-12h
Atenolol	0.5-1	2 (max: 100 mg/day)	12-24h
Furosemide	0.5-2	6	4-12h
Hydrochlorothiazide	1	3 (max: 50 mg/day)	12h
Spironolactone	1	3.3 (max: 100 mg/day)	6-12h
Clonidine( ≥12 years)	0.2 mg/day	2.4 mg/day	12h
Prazosin	0.05-0.1	0.5	8h
Hydralazine	0.75	7.5 (max: 200 mg/day)	6h
Minoxidil			
< 12 years	0.2	50 mg/day	6-8h
≥ 12 years	5 mg/day	100 mg/day	

max: maximum; h: hour.

## Hypertensive crisis

Hypertensive emergency is characterized by acute BP elevation associated with TOD, which can comprise neurological, renal, ocular and hepatic impairment or myocardial failure, and manifests as encephalopathy, convulsions, visual changes, abnormal electrocardiographic or echocardiographic findings, and renal or hepatic failure.^34^ Hypertensive urgency is described as BP elevation above the 99^th^ percentile plus 5 mm Hg (stage 2), associated with less severe symptoms, in a patient at risk for progressive TOD, with no evidence of recent impairment. Oral drugs are suggested, under monitoring, with BP reduction in 24-48 hours.^2^ In HE, the BP reduction should occur slowly and progressively: 30% reduction in the programed amount in 6-12 hours, 30% in 24 hours, and final adjustment in 2-4 days.^35^ Very rapid BP reduction is contraindicated, because it leads to hypotension, failure of self-regulating mechanisms, and likelihood of cerebral and visceral ischemia.^36^ The HE should be treated exclusively with parenteral drugs. In Brazil, the most frequently used drug for that purpose is SNP, which is metabolized into cyanide, which can cause metabolic acidosis, mental confusion, and clinical deterioration. Thus, SNP administration for more than 24 hours requires monitoring of serum cyanide levels, especially in patients with renal failure.^35,36^ After patient's stabilization with SNP, an oral antihypertensive agent should be initiated, so that the SNP dose can be reduced. The use of SNP should be avoided in pregnant adolescents and patients with central nervous system hypoperfusion.

Special clinical conditions can be managed with more specific hypotensive agents for the underlying disease. Patients with catecholamine-producing tumors can be initially alpha-blocked with phenoxybenzamine, or prazosin if the former is not available, followed by the careful addition of a BB. After BP control and in the absence of kidney or heart dysfunction, a sodium-rich diet is suggested to expand blood volume, usually reduced by the excess of catecholamines, favoring postoperative BP management and reducing the chance of hypotension. An IV short-acting antihypertensive drug should be used for intraoperative BP control. Furosemide is the first-choice drug for HC caused by fluid overload, for example, in patients with kidney disease, such as acute glomerulonephritis. In case of oliguria/anuria, other antihypertensive drugs can be used concomitantly, and dialysis might be necessary for blood volume control. Arterial hypertension associated with the use of cocaine or amphetamines can be treated with lorazepam or other benzodiazepine, which is usually effective to control restlessness and AH. In the presence of a HE, phentolamine, if available, is the drug of choice, and should be used in combination with lorazepam.^37^

Table 7 shows the most frequently used drugs in pediatric HE.^38,39^

**Table 7:** Major pediatric drugs and doses used to control hypertensive emergency^2^^,95,96^

Drug	Route	Dose	Action beginning	Duration
Sodium nitroprusside	IV	0.5-10µg/kg/min	Seconds	Only during infusion
Labetalol	IV	0.25-3 mg/kg/h or Bolus: 0.2-1 mg/kg followed by infusion: 0.25-3 mg/kg/h	2-5 min	2-4 h
Nicardipine	IV	1-3µg/kg/min	2-5 min	30 min-4 h, the greater, the longer the use
Hydralazine	IV IM	Bolus: 0.2-0.6 mg/kg IV, IM, max = 20 mg	10-30 min	4-12 h
Esmolol	IV	Attack: 100-500µg/kg followed by infusion: 50-300µg/kg/min	Seconds	10-30 min
Phentolamine	IV	Bolus: 0.05-0.1 mg/kg, max = 5 mg/dose	Seconds	15-30 min

IV: intravenous; IM: intramuscular; min: minute; h: hour.

## Figures and Tables

**Table:** 90^th^ percentile

SBP	87	101	106	106	106	106	106	106	106	106	106	106	106
DBP	68	66	63	63	63	66	66	67	68	68	69	69	69
Height (cm)	51	59	63	66	68	70	72	73	74	76	77	78	80
Weight (kg)	4	4	5	5	6	7	8	9	9	10	10	11	11

Source: Report of the Second Task Force on Blood Pressure Control in Children - 1987. Task Force on Blood Pressure Control in Children. National Heart, Lung and Blood Institute, Bethesda, Maryland. Pediatrics 1987;79(1):1-25.

**Table:** 90^th^ percentile

SBP	76	96	101	104	105	106	106	106	106	106	106	106	106
DBP	68	66	64	64	65	66	66	66	66	67	67	67	67
Height (cm)	54	56	56	56	61	63	66	68	70	72	74	75	77
Weight (kg)	4	4	4	5	5	6	7	8	9	9	10	10	11

Source: Report of the Second Task Force on Blood Pressure Control in Children - 1987. Task Force on Blood Pressure Control in Children. National Heart, Lung and Blood Institute, Bethesda, Maryland. Pediatrics 1987;79(1):1-25.
